# Selective Protective Potency of Yersinia pestis ΔnlpD Mutants

**Published:** 2015

**Authors:** S. V. Dentovskaya, S. A. Ivanov, P. Kh. Kopylov, R. Z. Shaikhutdinova, M. E. Platonov, T. I. Kombarova, T. V. Gapel’chenkova, S. V. Balakhonov, A. P. Anisimov

**Affiliations:** State Research Center for Applied Microbiology, Obolensk, Moscow Region, 142279, Russia; Irkutsk Antiplague Research Institute of Siberia and Far East, Trilissera Str., 78, 664047, Irkutsk, Russia

**Keywords:** Yersinia pestis, ΔnlpD mutant, selectivity of protective potency, live plague vaccine

## Abstract

It has recently been shown that the NlpD lipoprotein is essential to *Yersinia pestis *virulence and that subcutaneous administration of the *nlpD *mutant could protect mice against bubonic and pneumonic plague better than the EV vaccine strain [PLoS One 2009. V. 4. № 9. e7023]. In this study, similar Δ*nlpD *mutants were generated on the basis of other *Y. pestis *parent strains, including strains from the subspecies *microtus*, which is avirulent to guinea pigs and humans. Comparative testing confirmed that immunization of mice with Δ*nlpD* mutants induces immunity 105 times more potent than the one induced by the administration of the EV vaccine strain. At the same time, NlpD- bacteria failed to protect guinea pigs in the case of a subcutaneous challenge with* Y. pestis*, inducing a 106 times less potent protection compared with that conferred by immunization with the EV vaccine strain. The possible causes of the observed phenomena are discussed.

## INTRODUCTION

Live vaccines stimulate not only humoral, but also cell-mediated immunity, which, in some species, plays the leading role in the immunogenesis of plague [1-8]. Furthermore, live vaccines constructed on the basis of attenuated strains contain not merely one or two immunodominant antigens, but a whole range of complex (protein complexes with lipopolysaccharides (LPS), etc.) conformationally labile and minor antigens, which ensure induction of a “heterogeneous” immune response after a single immunization. This immune response can protect different species of animals against bacterial pathogens, including bacteria with partially altered antigenic specificity, in case of both subcutaneous and aerosol administrations [3, 7-11]. However, a commercial live plague vaccine created on the basis of the *Yersinia pestis *EV strain can cause local and systemic adverse reactions of varying severity in 5–29% of subjects with a normal immune status, regardless of route of administration [1, 2, 12, 13]. Therefore, studies aimed at creating live plague vaccines based on precisely attenuated strains of *Y. pestis *with superior immunogenicity and reduced reactogenicity compared to those of the commercial EV vaccine strain remain relevant [2, 8, 14-19].

Potential target genes for the attenuation of virulent strains are either selected (i) by random mutagenesis with individually labeled transposons [20], (ii) using reverse vaccinology techniques [21-23] or (iii) chosen by investigating analogs of genes from other bacterial pathogens, whose mutations had been previously shown to reduce virulence [24]. For example, a relationship has been established in the past decade between the expression of the *nlpD/lppB *(novel lipoprotein D/lipoprotein B) family of genes and survival of some gram-negative bacteria in a stressful environment, as well as their pathogenicity [18, 25, 26]. It has been shown [14] that lipoprotein NlpD is essential for virulence of the plague pathogen *Y. pestis *in case of subcutaneous and aerosol administration. Moreover, immunization of mice by 10^5^ CFU of Δ*nlpD*-mutant of* Y. pestis *Kimberley53 strain, followed by administra tion of 10^5^ LD_50_ of the wild-type Kimberley53 strain (1 LD_50_ = 1–3 CFU) resulted in a 100% survival rate, whereas the EV vaccine strain protected only 10% of the animals against death.

The purpose of this study was to construct Δ*nlpD* mutants of other parental *Y. pestis *strains, including strains of subsp. *microtus*, which are avirulent for guinea pigs and humans, and to evaluate their protective potency in mice and guinea pigs.

## MATERIALS AND METHODS

**Bacterial strains **used in the study and their characteristics are listed in *Table 1*. Strains of *Y. pestis *and *Escherichia coli *were grown in liquid or solid Hottinger culture media (various batches prepared in the SRCAMB) and LB (1% tryptone, 0.5% yeast extract, 1% sodium chloride) at pH 7.2. Selection of cells containing recombinant plasmids was carried out in the media supplemented with antibiotics ampicillin (100 μg/mL), chloramphenicol (10 μg/mL), and polymyxin B (100 μg/mL). Strains of* Y. pestis *for the immunization and infection of animals were grown at 28 °C for 48 h.

**Table 1:** Characteristics of the microorganism strains used in the study

Strain	Characteristics	Source of the strain and/or reference*
*Y. pestis*		
EV NIIEG line	pFra^+^pCad^+^pPst^+^Δpgm (subsp. pestis bv. orientalis), vaccine strain	SCPM-Obolensk
231	pFra^+^pCad^+^pPst^+^Pgm^+^ (subsp. pestis bv. antiqua), wild type	SCPM-Obolensk
231ΔnlpD	ΔnlpD mutant of 231	CS
I-3455	pFra^+^pCad^+^pPst^+^Pgm^+^ (subsp. microtus, bv. altaica)**, wild type	CCIARISFE
I-3455ΔnlpD	ΔnlpD mutant of I-3455	CS
I-2359	pFra^+^pCad^+^pPst^+^Pgm^+^ (subsp. microtus, bv. altaica), wild type	CCIARISFE
I-2359ΔnlpD	ΔnlpD mutant of I-2359	CS
*E. coli*		
DH5α λpir	F^-^, λ^-^, recA1, endA1, gyrA96, thi-1, hsdR17(rK-, mK+), supE44, recA1	[27]
S17-1 λpir	thi pro hsdR^-^ hsdM^+^ recA RP4 2‑Tc::Mu-Km::Tn7(Tp^R^ Sm^R^ Pm^S^)	[28]

* SCPM-Obolensk, State Collection of Pathogenic Microorganisms and Cell Cultures of the State Research Center for Applied Microbiology and Biotechnology (Rospotrebnadzor); CCIARISFE, Culture Collection of the Irkutsk Antiplague Research Institute of Siberia and Far East (Rospotrebnadzor).

** Names of *Y. pestis* subspecies and biovars as proposed in [29].

**Mutagenesis**

*Y. pestis *mutants were constructed by homologous recombination with a recombinant plasmid pCVD442-Δ*nlpD*::*cat *based on the suicide vector pCVD442 [30], in which a portion of the cloned coding sequence of the *nlpD *gene (nucleotides 112-318) was replaced with the *cat *gene from pKD3 plasmid [31] (*Figure*).

**Figure:**
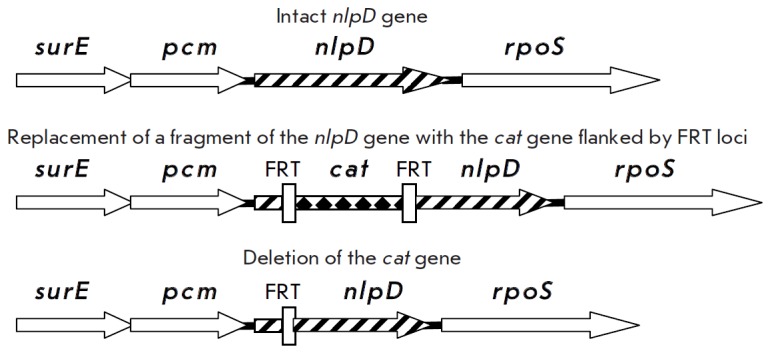
Construction of Y. pestis ΔnlpD mutants. Detailed description of the strategy is given in [30, 31]

pCVD442-Δ*nlpD*::*cat *plasmid from a donor *E. coli* S17-1 *λpir *strain was transferred into a recipient wild type *Y. pestis *strain (231, I-3455 or I-2359) by conjugation. Elimination of the suicide vector and selection of* Y. pestis *clones were performed in the presence of 5% sucrose and chloramphenicol [30]. The chloramphenicol resistance gene was removed using pCP20 plasmid [31] (*Figure*). pCP20 plasmid was removed by culturing bacteria at 40oC in a medium containing 2.5 mM of calcium chloride overnight. Clones that had lost resistance to the both ampicillin (100 μg/mL) and chloramphenicol (20 μg/mL) were selected. The accuracy of recombination was monitored by a polymerase chain reaction.

**Microscopic studies and bacteriological assays**

Microscopic studies, the rates of growth and lysis of cultures by plague bacteriophage L-413C, fibrinolytic and plasma-coagulase activities, pigmentation phenotype, and plasmid profile were assayed as described in [14, 32-34].

**Immunochemical studies**

F1 titers in *Y. pestis *strains under study were determined by a passive hemagglutination test as described in [35].

Antibody titers against F1 and LcrV antigens in the sera of animals immunized for evaluation of the immunity index (see below) were determined by indirect ELISA on day 21 after subcutaneous administration of the constructed and control strains. Antibody titers were determined individually in five randomly selected animals in each group of 40 animals immunized with one of the constructed or control strains, followed by calculation of the mean titer in the group. The titer value was defined as the highest dilution of specific antisera that corresponded to the optical density of the substrate solution at a wavelength of 492 nm, which was 0.1 higher than the value observed for the same dilution of the control [36]. The difference between the theoretical and experimental values of* A*_492_ was calculated and plotted versus appropriate dilutions of antisera, which were fitted by a polynomial function.

**Safety of *Y. pestis *strains**

The safety of the constructed *Y. pestis *strains in BALB/ c mice and guinea pigs was assessed as described in [35]. Cultures of *Y. pestis *strains under study were administered subcutaneously to mice (18–20 g) at a dose of 10^2^, 10^3^, 10^5^ and 10^7^ CFU (10 mice per dose) and five guinea pigs (180–200 g) at a dose of 1.5× 10^10^ CFU.

**Evaluation of immunogenic potency of vaccine candidates** was performed in accordance with the Methodological Guidelines [35]. The immunogenicity of the constructed strains was assessed by their ImD_50_ values. Guinea pigs (10 animals per group) were immunized subcutaneously in the upper third of the right femur by two-day-old agar cultures of the strains under study at doses of 4×10, 2×10^2^, 1×10^3^ and 5×10^3^ CFU in a total volume of 0.5 mL. BALB/c mice (10 animals per group) were immunized subcutaneously with 2×10^2^, 1×10^3^, 5×10^3^ and 2.5×10^4^ CFU in a total volume of 0.2 mL. The animals were challenged in the upper third of the left femur on day 21 after subcutaneous immunization at a dose corresponding to 200 DCL (LD_100_) of a virulent *Y. pestis *strain (in our experiments, 1 DCL was equal to 10 CFU in mice and 100 CFU in guinea pigs). Infected animals were kept under observation for 20 days. Animals that succumbed to infection were sacrificed and examined bacteriologically.

**The intensity of immunity (immunity index)**, i.e., the vaccine’s ability to protect animals against death after administration of high doses of virulent strains on day 21 after the immunization, was calculated using the following formula:

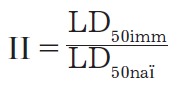



where II is the immunity index; LD_50imm_ is LD_50_ for animals immunized with a strain under study, CFU; and LD_50nai_ is LD_50_ for naive animals, CFU [35].

To determine the immunity index, the animals were immunized subcutaneously with two-day-old agar cultures of the constructed and control strains (40 guinea pigs and 40 mice per strain): guinea pigs at a dose of 5×10^3^ CFU in 0.5 mL, BALB/c mice at a dose of 104 CFU in 0.2 mL. On day 21 after the immunization, the animals were infected with a virulent *Y. pestis *231 strain at four doses: 10^2^, 10^4^, 10^6^, and 10^8^ CFU (guinea pigs in a volume of 0.5 ml, mice in a volume of 0.2 mL). Naive (control) animals were simultaneously infected at doses of 1, 5, 25, and 125 CFU in the same volume as the immunized ones. Infected animals were kept under observation for 20 days. Animals that succumbed to infection were sacrificed and examined bacteriologically.

**Statistical methods**

ImD_50_ values of *nlpD *strains and LD_50_ of the virulent strain for immunized and naive animals, as well as the corresponding confidence intervals (95% level of confidence), were calculated using the Karber method [37].

## RESULTS

**Construction and characterization of NlpDvariants of virulent *Y. pestis *strains**

231Δ*nlpD*, I-2359Δ*nlpD, *and I-3455Δ*nlpD *mutants without antibiotic resistance genes were obtained by site-directed mutagenesis of the *nlpD *gene in *Y. pestis* subsp. *pestis *strain 231 and two subsp. *microtus *bv. altaica strains I-2359 and I-3455, respectively, followed by deletion of the chloramphenicol resistance marker.

Microscope analyses of Gram-stained smears prepared from 231Δ*nlpD*, I-2359Δ*nlpD*, and I-3455Δ*nlpD* strains revealed that culturing of the mutant strains at 28°C results in the formation of undivided chains containing an average of 8.2±3.6 cells/chain as opposed to aggregative morphology of cultures of the parent *Y. pestis *231, I-2359, and I-3455 strains. Elevation of the culturing temperature to 37°C reduced the mean number of mutant cells per chain to 4±2.5 for Δ*nlpD *mutants. The morphology of cells and cell clusters of the wild-type strains was temperature-independent. The growth rate of *Y. pestis *231Δ*nlpD *was identical to that of the parent strain at both 28 and 37°C.

The constructed Δ*nlpD *mutants were lysed by the plague diagnostic bacteriophage L-413C. Based on the data of the passive hemagglutination test, the level of F1 capsular antigen in the mutants was 4–16 times higher than that in the culture of *Y. pestis *vaccine strain EV line NIIEG grown under similar conditions (1–4 μg/109 CFU and 0.25 μg/109 CFU, respectively). These Δ*nlpD*-mutants were not inferior to the EV strain in terms of their fibrinolytic and plasma coagulase activities. They contained the same three pFra, pCad, and pPst plasmids as the vaccine strain; however, they differed from the EV strain in their ability to absorb pigments.

**Determination of safety of the strains**

All strains of *Y. pestis *defective in the *nlpD *gene, 231Δ*nlpD*, I-3455Δ*nlpD, *and I-2359Δ*nlpD*, as well as* Y. pestis *EV vaccine strain, were avirulent in mice upon subcutaneous administration to BALB/c mice (100% survived the infection at a dose of 102, 103, 105 and 107 CFU), and in guinea pigs (100% survival rate at a dose of 1.5×10^10^ CFU). The animals were kept under observation for 50 days.

**Antibody response to vaccine candidates**

Levels of antibodies against *Y. pestis *F1 and LcrV in the blood of BALB/c mice were evaluated on day 21 after subcutaneous immunization with *Y. pestis *strain under study at a dose of 10^4^ CFU (*Table 2*). Mean antibody titers against F1 and LcrV in the mouse sera after vaccination with cultures of *Y. pestis *231Δ*nlpD* and I-3455Δ*nlpD *exceeded those obtained for *Y. pestis* I-2359Δ*nlpD *and EV vaccine strain (p < 0.05).

**Table 2:** Antibody response to administration of Y. pestis strains based on ELISA data

Mean IgG titers (inverse values)				
Strains	231ΔnlpD	I-3455ΔnlpD	I-2359ΔnlpD	EV NIIEG
Guinea pigs				
Antigen				
F1	4435 ± 1625	2650 ± 1045	130 ± 80	127630 ± 52830
LcrV	1555 ± 840	710 ± 260	920 ± 630	94390 ± 49290
Mice				
Antigen				
F1	942560 ± 16620	9140 ± 1590	550 ± 95	310 ± 140
LcrV	2465 ± 970	6715 ± 1620	1580 ± 850	235 ± 85

Titers of anti-F1- and anti-LcrV-antibodies in the blood of the vaccinated and control guinea pigs were determined on day 21 after subcutaneous immunization with *Y. pestis *strain under study at a dose of 5×10^4^ CFU (*Table 2*). According to our data, mean antibody titers against F1 and LcrV in the sera of guinea pigs after administration of the EV vaccine strain were two– three orders of magnitude higher than the values for the strains 231Δ*nlpD*, I-3455Δ*nlpD, *and I-2359Δ*nlpD* (*p * < 0.05). Antibody response to *Y. pestis *F1 and LcrV in guinea pigs after administration of the vaccine and constructed strains varied; in mice, the response was more uniform.

The levels of circulating anti-F1 and anti-LcrV antibodies in the blood of mice immunized with the vaccine-candidate strains *Y. pestis *231Δ*nlpD *and I-3455Δ*nlpD *were significantly higher than those for guinea pigs immunized with the same strains.

In the control group, no antibodies against *Y. pestis* F1 and LcrV were detected after administration of an isotonic sodium chloride solution.

**The protective efficacy of vaccine candidate strains**

The indicators of immunogenic potency and immunity indices for BALB/c mice after a single immunization are presented in *Table 3*. For laboratory animals of this species, ImD_50_ of *Y. pestis *231Δ*nlpD *and I-3455Δ*nlpD* strains was 58 and 26 times lower than that of the EV vaccine strain, respectively; however, the value was 1.5 times higher for I-2359Δ*nlpD *strain. The immunity indices for *Y. pestis *231Δ*nlpD *and I-3455Δ*nlpD *were five orders of magnitude higher than that of the EV vaccine strain, but they were only 2.5 times higher for I-2359Δ*nlpD*.

**Table 3:** Indicators of immunogenic potency and intensity of immunity in BALB/c mice vaccinated with *nlpD* mutants of *Y. pestis* strains 231, I-3455, and I-2359

Immunizing strain of *Y. pestis*	ImD_50_, CFU	Immunity index	
		LD_50_ upon challenging with *Y. pestis* 231, CFU	II
231ΔnlpD	1.3 × 10^2^ (5.3 × 10 ÷ 3.4 × 10^2^)	3.9 × 10^8^ (too large)	7.1 × 10^7^
I-3455ΔnlpD	2.9 × 10^2^ (1.2 × 10^2^ ÷ 7.5 × 10^2^)	2.5 × 10^7^(1 × 10^7^ ÷ 3.9 × 10^8^)	4.5 × 10^7^
I-2359ΔnlpD	1.1 × 10^4^ (4.4 × 10^3^ ÷ 2.8 × 10^4^)	2.5 × 10^3^ (6.3 × 10^2^ ÷ 3.9 × 10^3^)	4.5 × 10^2^
EV NIIEG	7.5 × 10^3^ (2.4 × 10^3^ ÷ 5.9 × 10^4^)	1.0 × 10^3^ (2.5 × 10^2^ ÷ 3.9 × 10^3^)	1.8 × 10^2^

The opposite was observed for guinea pig models in immunogenic potency and immunity index experiments (*Table 4*). ImD_50_ was 140, 66, and 1692 times higher for* Y. pestis *231Δ*nlpD*, I-3455Δ*nlpD, *and I-2359Δ*nlpD* strains, respectively, than for the EV vaccine strain. The immunity index of the EV vaccine strain was six orders of magnitude higher than that of the strains 231Δ*nlpD* and I-3455Δ*nlpD *and seven orders of magnitude higher than that of the strain I-2359Δ*nlpD.*

**Table 4:** Indicators of immunogenic potency and intensity of immunity in guinea pigs vaccinated with *nlpD*-mutants of *Y. pestis* strains 231, I-3455, and I-2359

Immunizing strain of *Y. pestis*	ImD_50_, CFU	Immunity index	
		LD_50_ upon challenging with *Y. pestis* 231, CFU	II
231ΔnlpD	9.1 × 10^3^ (too large)	63 (1.6 × 10 ÷ 2.5 × 10^2^)	3.7
I-3455ΔnlpD	4.3 × 10^3^ (too large)	158 (4.0 × 10 ÷ 6.3 × 10^2^)	9.3
I-2359ΔnlpD	1.1 × 10^5^ (too large)	10 (3 ÷ 4.0 × 10)	0.59
EV NIIEG	65 (1.6 × 10 ÷ 2.6 × 10^2^)	1.6 × 10^8^ (too large)	9.4 × 10^6^

## DISCUSSION

To evaluate the universal applicability of a combination of attenuation and high immunogenicity of *Y. pestis* Δ*nlpD *mutants, site-directed mutagenesis was performed in three wild-type *Y. pestis *strains: one subsp.* pestis *bv. antiqua strain 231 and two subsp. *microtus* bv. altaica strains I-3455 and I-2359. Subsp. *microtus* strains, which include biovar altaica [29], are known to be virulent for mice, but avirulent for guinea pigs, rabbits, and humans [38, 39]. It is believed [40] that subsp.* microtus *strains possessing all protective antigens are avirulent for humans and can be used to design live plague vaccines. Furthermore, one of the strains used in our study, bv. altaica I-3455, produces LcrV with increased immunogenic/protective activity (due to the replacement of tryptophan at position 113 with glycine) [41].

In the Russian Federation all trials of attenuated *Y. pestis *vaccine candidate strains are conducted by comparing them to the reference *Y. pestis *vaccine strain EV line NIIEG. According to [35], “the strain, proposed as a vaccine, must match or surpass the reference vaccine strain in immunogenicity, match the control strain in safety and reactogenicity or be safer; however, some non-essential characteristics that define it as a member of *Y. pestis *species may be different from the reference strain.” “Non-essential characteristics” mean that “an experimental vaccine candidate strain must:

– be susceptible to the plague diagnostic bacteriophage L-413C;

– have typical culture-morphological properties;

– have F1 titer not lower than that obtained for the culture of the control *Y. pestis *EV strain, grown under similar conditions;

– have less than 0.3% calcium-independent mutants in the population of plague microbe cultures, which has been passaged through laboratory animals and exposed to neither long-term storage nor physical impact;

– at least match the fibrinolytic and plasma coagulase activities of the control strain;

– constructed and control strains must have pigmentation- negative phenotype; and

– the vaccine strains under study must have the same electrophoregram pattern as the reference EV strain: three bands of DNA plasmids corresponding to pFra (60 MD), pCad (47 MD), and pPst (6 MD)”.

The first of the plasmids encodes the main *Y. pestis *immunogen, its capsular F1 antigen. The second one encodes a system that allows extracellularly located bacteria to neutralize the host cells involved in the immune response, Yop virulon, and the second immunodominant antigen LcrV involved in the virulon system; the third plasmid encodes the plasminogen activator responsible for dissemination of the plague microbe in host tissues [13].

The constructed *Y. pestis *Δ*nlpD *mutants met most of the requirements for non-essential indicators of plague microbe vaccine strains [35]. They were susceptible to L-413C bacteriophage, the production of F1 in the mutant strains was 2–4 times higher than that in the EV strain, fibrinolytic and plasma coagulase activity in all strains were at the same level, and all strains contained a full set of the three classic *Y. pestis *plasmids.

The culture-morphological properties of Δ*nlpD* mutants of the 231, I-3455, and I-2359 strains, such as their filamentous morphology, distinguish them from wild-type bacteria and the EV vaccine strain, which is in agreement with data [14] indicating that *Y. pestis* NlpD lipoprotein plays an important role in cell separation. Particular features of cell separation may be the main cause of attenuation in Δ*nlpD *mutants.

The constructed strains preserved their ability to absorb pigments at the level of the wild-type strains, since their attenuation did not result from deletion of the *pgm *locus, but rather that of the *nlpD *structural gene.

In terms of compliance of Δ*nlpD *mutants with the main selection criteria for *Y. pestis *vaccine strains, the degree of attenuation (safety) of NlpD- strains was not inferior to that of the EV stain in mice and guinea pigs. However, the second criterion, immunogenicity, was more ambiguous. This parameter was evaluated in two animal species in three independent tests: titers of antibodies against F1 and LcrV, determination of immunizing doses, which protect 50% of infected animals against death, and immunity indices.

Even though the antibody levels are only partially correlated with the protective efficacy of plague vaccines, the humoral immunity plays an important role in protection against the disease [42]. The data obtained demonstrate the development of an effective immune response in mice after administration of attenuated *Y. pestis *cultures; the Δ*nlpD *strains were statistically significantly superior to the EV vaccine strain. The opposite was observed in the experiments on guinea pigs; the vaccine strain was superior to Δ*nlpD *mutants in its ability to induce an antibody response.

In a mouse model, *Y. pestis *strains 231Δ*nlpD *and I-3455Δ*nlpD *were statistically significantly superior to the EV strain in terms of ImD_50_ and, especially, II values. In experiments on guinea pigs, the constructed strains were inferior to the vaccine strain and the immunity index in animals immunized with Δ*nlpD *mutants was close to 1; i.e., it almost did not differ from this index in naive animals.

The results of our experiments confirm the findings of other researchers showing that different animal species have different reactions to the same antigen/vaccine formulations [12, 43-48]. The differences in the protective efficacy of *Y. pestis *NlpD^-P^ mutants in guinea pigs and mice may be attributed to the peculiarities of immunogenesis in these biological models [2]. The lack of protective efficacy of Δ*nlpD *mutants in guinea pigs can have at least two possible explanations.

On the one hand, attenuation by mutation in the* nlpD *gene may result in an excessive decrease in residual virulence [12, 49], and, therefore, the mutants are unable to replicate in the guinea pigs for a period of time long enough to induce immunity.

On the other hand, it is possible that NlpD lipoprotein of the plague pathogen is the insoluble “residual” antigen R or one of its constituents and that it induces potent long-term protection against the plague in guinea pigs [50-52]. Consequently, its absence in the cultures used for immunization may be the main reason for the weak protective properties of Δ*nlpD *mutants.

We are currently conducting experiments to test these two hypotheses.

## CONCLUSIONS

To sum up the data obtained in this study, without additional modifications that would increase their immunogenicity in guinea pigs, Δ*nlpD *mutants are not promising candidates for live plague vaccines due to the selectivity of their protective potency in different animal species.

## Abbreviations

SCPM-Obolensk: State Collection of Pathogenic Microbes and Cell Cultures on the base of State Research Center for Applied Microbiology and Biotechnology
SRCAMB: State Research Center for Applied Microbiology and Biotechnology
II: index of immunity
ELISA: enzyme-linked immunosorbent assay
CFU: colony-forming unit
LPS: lipopolysaccharide
CS: current study
CCIARISFE: Culture Collection of Irkutsk Antiplague Research Institute of Siberia and Far East
PHAT: passive hemagglutination test
DCL (LD100): absolutely lethal dose (dosis certa letalis)
ImD50: immunizing dose protecting 50% of infected animals from death
LB: Luria-Bertani broth
LD50: dose lethal for 50 % of infected animals
